# Building organizational reputation in the European regulatory state: An analysis of EU agencies' communications

**DOI:** 10.1111/gove.12438

**Published:** 2019-08-18

**Authors:** Dovilė Rimkutė

**Affiliations:** ^1^ Leiden University

## Abstract

Organizational‐reputation literature has advanced our understanding about the U.S. regulatory state and its agencies. However, we lack contributions on what a reputational account can add to our knowledge about the European regulatory state, the strategic behavior of supranational agencies, and their endeavors to legitimize themselves in a multilevel political system. We know little of how reputation‐management strategies vary across EU agencies and why. The study offers the very first mapping of organizational‐reputation‐management patterns across all EU agencies, as well as the first empirical assessment on how reputational considerations guide supranational agencies' legitimation strategies. The results indicate that EU agencies facing higher reputational threats revert to their avowed raison d'être (i.e., technical conduct). We find that regulatory agencies utilize a more diverse set of reputational strategies by emphasizing the technical, procedural, and moral reputations more than nonregulatory agencies, whereas social‐policy agencies foster their technical reputation more than economic‐policy agencies.

## 1 INTRODUCTION

Organizational reputation scholarship offers a novel research agenda focusing on the development of the regulatory state and the strategic behaviors of government agencies. Although the bureaucratic reputation theory has been mostly applied to the context of the U.S. and Israeli regulatory states (Carpenter, 2001, 2010; Gilad, Maor, & Bloom, 2013; Maor, 2011; Maor & Sulitzeanu‐Kenan, 2013, 2015; Maor, Gilad, & Bloom, 2012), we lack scholarly contributions on organizational reputation literature's insight on the European regulatory state, the strategic behavior of supranational organizations, and their attempts to legitimize their roles in a multilevel regulatory polity. To that end, this study brings together organizational reputation and EU governance literature to explore how bureaucratic reputation is built in a European regulatory state by its core regulatory actors—that is, EU agencies—and explain the variation of reputation‐management strategies on which supranational agencies draw to communicate their roles and activities to relevant audiences.

Such contribution is relevant because one of the key features of the rise of the regulatory state in Europe is the creation of quasiautonomous EU agencies (Majone, 1994). The transfer of regulatory powers has witnessed not only a “sideways” move to nonmajoritarian institutions but also a move at the EU level (Lodge, 2008). To date, 45 EU agencies and bodies contribute to the European regulatory state. Although agencies vary in the way they have been designed in diverse regulatory domains (Busuioc, 2013; Wonka & Rittberger, 2010), it is evident that EU agencies are at the heart of the European regulatory state.

The study draws on the definition of organizational reputation introduced by Carpenter (2010) and adds a more refined conceptualization and operationalization on the kinds of reputation‐management strategies that are being used by agencies to secure their organizational reputation. Carpenter argued that agencies can cultivate their reputations in several ways: by creating a superior track record in terms of their technical or scientific conduct (*technical reputation*), by demonstrating their capacity to make significant regulatory changes and take effective actions (*performative reputation*), by engaging in procedurally fair activities (*legal‐procedural reputation*), or by emphasizing the wider moral implications of their activities (*moral reputation*). However, we have very limited knowledge about which aspects of organizational reputation are relevant to supranational authorities and what factors explain the variance across agencies. To that end, the core research questions of this study are the following: How does the repository of reputation‐cultivation strategies vary across EU agencies? Do reputational considerations account for the diverse reputation‐management strategies across agencies?

To approach these research questions theoretically, this study tailors the core concepts and arguments of reputation literature to EU governance scholarship and, in so doing, offers reputation‐based explanations accounting for the variation of reputational repertoire that supranational agencies employ to engender the belief vis‐à‐vis relevant audiences that they can carry out the functions entrusted to them by national governments in a professional, efficient, procedurally appropriate, and/or ethical way. Such contribution is pertinent because the potential of bureaucratic reputation theory for the European regulatory state and EU agencies has been largely neglected despite its promising research agenda offering novel concepts and explanations about reputation‐promotion and blame‐avoidance games that supranational agencies may play to legitimize themselves vis‐à‐vis the multiplicity of audiences observing and judging their outputs, processes, and behavior.

More specifically, the study introduces and examines two sets of expectations. First, it draws on the *reputation uniqueness* concept—which refers to an agency's responsibility to deliver outputs and outcomes that cannot be provided by any other organization in the entity (Carpenter, 2001, 2010; Maor & Sulitzeanu‐Kenan, 2015)—to argue that variation in reputation‐management strategies across agencies can be explained by different agency roles, competencies, and mandates. This is the case because the maintenance and enhancement of agencies' unique reputation depend on their functions and actions being widely acknowledged on the basis of their distinct character that agencies are assigned with at the moment of their establishment (Carpenter, 2010; Carpenter & Krause, 2012). Second, a reputational account suggests that agencies' behavior is determined not only by its reputation uniqueness but also by *reputational threats* originating from the multiplicity of conflicting audiences observing and judging the outputs and/or processes of agencies (Carpenter, 2010; Gilad, 2008, 2012; Gilad et al., 2013; Maor et al., 2012). Carpenter (2010) suggested, “when trying to account for a regulator's behavior*, look at the audience, and look at the threats* [italics in original]” (p. 832). As a result, reputational threats are expected to urge agencies to cultivate their intermediate responses carefully. Agencies as strategic actors are expected to do their utmost to produce different blame‐avoidance reactions (Maor & Sulitzeanu‐Kenan, 2013; Weimer, 2006).

The empirical analysis of this study rests on a quantitative content analysis of EU agencies' communications. The study examines not only how EU agencies validate their roles and activities to the most relevant stakeholders but also what they choose to emphasize. This research strategy provides us with a more differentiated understanding of how organizational reputation is built within the European regulatory state. It also informs about EU agencies' efforts to build positive reputations vis‐à‐vis relevant constituencies and whether EU agencies' self‐presentation (as observed in their communications) is in line with the core features of the EU regulatory state that exclusively emphasize expert‐based and results‐oriented legitimation sources or, in contrast, whether external reputational threats make EU agencies drift away from their avowed raison d'être by being receptive to external expectations.

The study makes several novel contributions to the scholarship of organizational reputation and EU governance. First, in recent years, interest in regulatory communication and the reputation theory has grown (Gilad et al., 2013; Maor et al., 2012); however, only lately have these aspects started to be explored in an EU regulatory context (Busuioc & Rimkutė, 2019a, 2019b). This article offers the first empirical assessment of all 45 EU agencies' reputation‐management strategies that supranational authorities employ to legitimize their regulatory roles in the European regulatory state. This contribution is relevant because, although the agency phenomenon has received much scholarly attention, scholars in the field have only explored the reputational aspects of EU agencies to a limited extent (e.g., Busuioc, 2015; Rimkutė, 2018). Furthermore, research on the EU agencies is marked by an exclusive focus on a small‐n/single‐case analysis, leaving the following questions unanswered: How do EU agencies compare to each other? Do they employ diverse strategies to build strong reputations and which factors account for these differences? To that end, a quantitative study relying on a novel measurement of the four reputation‐management dimensions opens many avenues for future research by mapping out the reputation‐management strategies of all supranational agencies.

Second, the focus on supranational agencies, in turn, provides novel insights into the theoretical development of bureaucratic reputation theory by exploring its potential to account for the behavior of *supranational public organizations* that operate in a complex multilevel governance system. Such setting is marked by the multiplicity of conflicting audiences, complex institutional arrangements, and severe legitimacy‐ and authority‐related issues. More specifically, supranational agencies possess multiple and diverse roles, duties, and mandates as they are established on a case‐by‐case basis (Chiti, 2013). They operate in high institutional complexity as they possess multiple formal superiors. For instance, supranational agencies have to depend on the European Commission (EC) and the European Parliament (EP) to draft regulations, as well as on the willingness of national authorities to cooperate in implementing and policing the enforcement of supranational rules (Busuioc & Rimkutė, 2019a). Furthermore, EU agencies are exposed to wider informal networks compared to their national counterparts. This suggests that the day‐to‐day activities of supranational authorities are observed by manifold—and usually conflicting—constituencies, which, in turn, require EU agencies to engage in a diverse set of reputation‐balancing strategies to deal with incompatible expectations. Such setting allows to fully exploit the conceptual richness of bureaucratic reputation theory that emphasizes the explanatory relevance of reputation uniqueness (i.e., the unique and distinct mandates and functions exclusively entrusted to agencies) and the context in which public organizations operate (i.e., reputational threats originating from the multiplicity of conflicting audiences judging the regulatory outputs and/or processes of agencies). Against this background, the study introduces and tests novel theoretical propositions specifying how reputation uniqueness and reputational threats guide agencies' reputation‐management strategies aimed at legitimizing themselves vis‐à‐vis multifaceted audiences.

## 2 ORGANIZATIONAL REPUTATION

Organizational‐reputation scholarship notes that the successful endeavors to foster good organizational reputations have numerous benefits to government agencies (Carpenter, 2010; Carpenter & Krause, 2012; Gilad, 2008, 2009; Maor & Sulitzeanu‐Kenan, 2013). The effective management of reputation may increase agencies' autonomy from political superiors and/or even buffer the agency from exterior accusations and attacks (Carpenter, 2010; Carpenter & Krause, 2012; Maor et al., 2012). Furthermore, it can result in increased regulatory authority that exceeds agencies' legal mandates. For instance, an agency can start engaging in a “self‐consistent action that neither politicians nor organized interests prefer but that they either cannot or will not overturn or constrain in the future” (Carpenter, 2001, p. 17). Finally, fruitful efforts to build good reputations may bring broad public support and even contribute to the legitimation of regulatory powers because “[l]egitimacy is…a product of successful reputation management by selectively responding to various reputational threats” (Rimkutė, 2018, p. 72).

As argued previously, possessing a good organizational reputation is a valuable organizational asset. To that end, bureaucracies are deemed to engage in extensive efforts to obtain one. Government agencies carefully construct their communications with external audiences to shape the way in which their multiple audiences judge their roles and activities (Gilad et al., 2013; Maor et al., 2012). Given that they operate in settings marked by competing interests, organizations selectively respond “to reputational threats that can potentially harm their distinctive organisational reputation” (Rimkutė, 2018, p. 72; see also Gilad, 2009; Gilad et al., 2013; Maor et al., 2012). In other words, as agencies are observed and judged by diverse—and often clashing—external audiences, they have to play different reputation–promotion games: “They [agencies] have a repository of ideas, values, and strategies that they may combine in various ways, deploy them politically and redeploy them between different audiences, thereby redefining relations with these audiences” (Maor, 2015, p. 10). To pursue positive reputations, agencies may have to adjust their communications to be in line with the expectations and preferences of their most relevant audiences. They may strategically emphasize specific aspects of their day‐to‐day activities to change the perceptions of relevant audiences.

However, obtaining and maintaining a good reputation are exceedingly difficult undertakings because organizational reputation is a multidimensional concept, making it challenging for organizations to find the right balance between the diverse aspects and combinations of organizational‐reputation types. Carpenter (2010) noted that a public organization does not possess a positive reputation per se; it can have a positive reputation for its professional rigorousness (technical reputation), capacity to deliver effective outputs and outcomes (performative reputation), commitment to due processes (legal–procedural reputation), or engagement in ethical behavior and safeguarding of the most prevalent values (moral reputation) (see Table 1). For a more elaborated description of reputational dimensions see Carpenter (2010).

**Table 1:** Summary of organizational reputation dimensions

Reputation dimensions	Signals that the agency sends to audiences	Focus of agency communication
Technical	Agency sends strong professional and technical signals	Scientific accuracy, soundness of (scientific) evidence, methodological quality, rigorous evidence‐selection criteria
Performative	Agency emphasizes its ability to attain goals set in its formal mandate	Effective and efficient regulatory outputs and outcomes
Legal‐procedural	Agency emphasizes a thorough engagement in socially acknowledged procedures	Due process, adherence to socially approved and fair procedures
Moral	Agency signals its commitment to wide moral implications and the ethical aspects of its conduct	Compassion to those affected by agency decisions, transparency, inclusiveness, openness, fairness, accessibility

The multiplicity of reputation‐balancing strategies generates a considerable latitude for agencies in preserving and cultivating their reputations. Agencies can simultaneously play different reputation–promotion games. They can select various dimensions on which they focus when conducting their core tasks and duties (Carpenter & Krause, 2012). The successful management of organizational reputation entails finding the right balance between technical, procedural, performative, and moral reputation‐management strategies to address the most important expectations and fight threatening reputational risks. However, we know little of how the four organizational traits are combined, as well as which of them prevail in the European regulatory state and across the diverse set of EU agencies that possess diverse reputation uniqueness and face different degrees of reputational threats.

## 3 ORGANIZATIONAL REPUTATION IN THE EUROPEAN REGULATORY STATE

Because the European regulatory state has limited capacities to engage in activities that are common to welfare states (i.e., redistributive social policies), from the outset, it has grown instead in executive terms by increasing its influence through a wide array of regulatory activities: “Lacking an independent power to tax and spend, the EC/EU had no alternative but to develop as *an almost pure type of regulatory state*” (Majone, 1999, p. 2, emphasis added). The European regulatory state is deemed to follow a consequentialist perspective and political rule that is legitimized with reference to the efficacy and effectiveness of its attained goals and ends (Scharpf, 1999). In this form of legitimation—also known as output/performance‐oriented or results‐based legitimacy—public organizations legitimize themselves by emphasizing the delivery of effective solutions to common issues.

Majone further argued that the rapid processes of agencification and the growing role of EU‐level regulators were formally sustained by referring to “the need to achieve credible policy commitments” (1999, p. 2). All EU agencies share one important aspect that is at the core of their reputation uniqueness: A core rationale for the creation of independent EU regulators is a plea for a result‐oriented and expertise‐based approach to tackle the common concerns of Member States. EU agencies are created to function as independent bodies that can support EU institutions with sound evidence that lead to effective results. They are expected to deliver technical solutions, as well as provide reliable information and scientific expertise.

Because the legitimacy of EU agencies “depends on their capacity to engender and maintain the belief that they are the most important ones for the functions entrusted to them” (Majone, 1999, p. 7), one should expect the core EU agencies' legitimation efforts to be focused on output‐oriented legitimacy (as opposed to input‐oriented legitimacy). In other words, EU agencies are expected to predominantly focus on cultivating their technical and performative reputations in their communications directed at external audiences. On the contrary, EU agencies are anticipated to spend less time and effort in harvesting the procedural and moral aspects of their organizational reputation because they are not regarded as their primary competence and does not feature the core rationale behind the creation of EU agencies.

Against this background, reputation literature has a high potential to enrich EU governance literature (that mainly draws on output‐legitimacy reasoning and anticipates EU agencies to focus on systematically conveying technical and performative signals to external audiences) by offering a more differentiated perspective on how supranational agencies legitimize themselves and under what conditions reputation‐management strategies are expected to vary across agencies. Namely, organizational literature suggests that reputation uniqueness and reputational threats can explain how agencies engage in reputation‐building endeavors to build and cultivate a positive organizational image. First, according to Carpenter, organizational reputation is “a set of symbolic beliefs about *the unique or separable capacities, roles, and obligations of an organization*, where these beliefs are *embedded in audience networks*” (2010, p. 45, emphasis added), suggesting that varying agency capacities, roles, and obligations (i.e., *possessing different reputation uniqueness*) are anticipated to account for how agencies manage their organizational reputation. Second, a reputational perspective argues that agency reputation‐management strategies are not curved in stone; on the contrary, they vary depending on *the degree of reputational threats* originating from the array of disagreeing audiences assessing the outputs, processes, and behavior of agencies (Carpenter, 2010; Gilad, 2008; Gilad et al., 2013; Maor et al., 2012). In the remainder, we introduce and further theorize how reputation uniqueness and reputational threats affect the reputation‐management strategies used by supranational agencies to legitimize themselves in the eyes of pertinent constituencies.

### 3.1 Reputation‐based explanations of EU agencies' communications

Upon their establishment, agencies are ascribed with a unique reputation. Reputation uniqueness is defined as the aptitude of an agency to deliver outcomes that cannot be provided by any other organization in the entity; that is, the agency has a responsibility to engage in the tasks that belong exclusively to them (Carpenter, 2010; Maor & Sulitzeanu‐Kenan, 2015). The development and upholding of agency's inimitable reputation depend on its roles and actions being widely approved on the basis of its distinct tasks, competences, and mandates (Carpenter, 2010; Carpenter & Krause, 2012). To that end, reputation uniqueness has to be cultivated by agencies by shaping their reputation‐balancing and protection strategies (Maor, 2011; Maor et al., 2012). Against this backdrop, the expectation is that agencies possessing diverse reputation uniqueness will focus on communicating different reputational traits.

More specifically, EU agencies vary in terms of their *roles*, *tasks*, and *mandates*. The creation of EU agencies follows two delegation logics: credibility‐ *or* efficiency‐based logic (Majone, 2001). Agencies created to solve credible‐commitment problems act as trustees (as opposed to agents) and, therefore, are assigned with far‐reaching responsibilities and higher independence from their superiors compared to agencies established for the efficiency‐based purposes. EU agencies' scholarship have provided evidence that *regulatory agencies* are more independent than their nonregulatory counterparts and are argued to tap into the credibility‐based logic of delegation (Wonka & Rittberger, 2010). On the contrary, *nonregulatory agencies* (i.e., executive and coordination) are more dependent on their political superiors and mainly exercise powers that are delegated to improve efficiency (e.g., to reduce the workload of the EC). To that end, nonregulatory agencies in the EU are in charge of concrete duties assisting the more efficient policy/decision making of EU institutions and Member States (e.g., clearly defined coordination and implementation‐specific tasks). Against this backdrop, we expect nonregulatory agencies to emphasize the performative aspects of their organizational reputation more than regulatory agencies because they were created to follow efficiency‐based principles that are at the heart of the performative dimension.

On the contrary, EU regulatory agencies possess tasks and responsibilities that are intended to enhance the credibility of a policy commitment (Majone, 2001). Nonetheless, despite their far‐researching regulatory responsibilities (compared to their nonregulatory counterparts), EU regulatory agencies face serious legal restrictions (compared to their national counterparts); that is, they often lack coercive powers that are based on a command‐and‐control principle (Chiti, 2013). As a result, EU regulatory agencies have developed the *information/persuasion‐based modes of regulation*: “the statutory limitations imposed on them [EU agencies] could provide a powerful incentive to agency executives to do something that is foreign to the traditional ‘corporate culture’ of fully fledge regulatory bodies, namely to develop systematically a regulatory approach, *primarily based on information and persuasion*” (Majone, 1997, p. 274, emphasis added). This, in turn, suggests that one should anticipate EU regulatory agencies to be more strategic than their nonregulatory counterparts. We, therefore, expect EU regulators to simultaneously play different reputation‐promotion games by focusing more not only on technical but also on the procedural and moral reputational‐management strategies compared to nonregulatory agencies.

We expect this because, when referring to EU‐level regulatory agencies acting as fiduciaries enhancing a credible commitment, the emphasis on the technical dimension is overly pronounced. For instance, regulatory agencies' “duties are deemed to be a highly scientific pursuit, predominantly rooted in the technical‐instrumental use of scientific knowledge and technical data” (Rimkutė, 2018, p. 70). The emphasis is on the evidence‐based solutions in which regulators rely upon the authority of experts: “Regulation depends so heavily on scientific, engineering and economic knowledge…expertise has always been an important *source of legitimization of regulatory agencies*” (Majone, 1997, p. 157, emphasis added). Thus, we expect EU regulatory agencies, to focus on the technical dimension more than their nonregulatory counterparts as the delivery of expertise‐based outputs is at the core of regulatory agencies' reputation uniqueness.

Furthermore, given their statutory limitations, EU regulators are expected to emphasize not only their information‐based regulatory tasks but also engage in the persuasion‐based mode of regulation by drawing on input‐based legitimation sources (i.e., focus on the legal‐procedural and moral reputation‐management strategies). In view of their complex legal status, EU regulatory agencies need to defend their professional integrity and independence: “unprofessional or politically motivated behaviour would compromise [EU regulatory agencies'] international reputation” (Majone, 1997, p. 262). To that end, focusing on the legal‐procedural dimension can be a good strategy to deal with multifaceted audiences in fields marked by technical complexity. This is the case because regulatory outputs are said to be appropriate if they arrive through a deliberative process that allows everyone likely to be affected to observe whether the procedures meet the requirements (Tyler, 2006). Decisions that have been made following a procedure that is perceived to be fair are more likely to be regarded as acceptable and receive support from external audiences. In a similar vein, given the need to engage in the persuasion politics, regulatory agencies are expected to cultivate their moral reputation more than nonregulatory agencies because regulatory outputs are believed to be acceptable if they correspond to prevailing values and if the agency manages to demonstrate its commitment to core ethical standards (e.g., fairness, inclusiveness, openness, transparency, good governance) (Carpenter, 2010).

Hypothesis 1a *Nonregulatory agencies are more likely than agencies assigned with regulatory tasks to focus on performative reputation*.

Hypothesis 2a/2b/2c *Regulatory agencies are more likely to focus on technical/procedural/moral reputation compared to agencies assigned with nonregulatory tasks*.

Following the argument of reputation uniqueness, social‐ and economic‐policy agencies are anticipated to focus on diverse reputation‐management strategies.^1^ We expect that agencies contributing to social policies are expected to harvest the moral and technical aspects of their organizational reputation, whereas their economic counterparts are more likely to focus on performative and procedural reputation because their core goal is to foster the EU economy by delivering outputs that increase the effectiveness and efficiency of European single market.

More specifically, the moral implications of social‐policy agencies' outputs are at the heart of their reputation uniqueness; therefore, moral dimension is anticipated to be cultivated more carefully by agencies contributing to social policies. However, EU regulatory state agencies contributing to social policies are formally required not only to protect EU consumers from unacceptable risks (e.g., health safety, environmental protection) but also deliver solutions that are exclusively based on reliable technical data and scientific knowledge (Rimkutė, 2018). At the core of social agencies' mandates is the obligation to draw on the best available science to provide the highest protection standards for EU citizens (Majone, 1996). Majone argued, “It is by now a truism that public policy is increasingly dependent on relevant, timely and, especially, credible information. Nowhere is such dependence stronger than in the area of social regulation—environmental and consumer protection, risk regulation, occupational health—where policy‐maker often faces problems at the frontier of scientific and technical knowledge” (1997, p. 264). Against this background, we expect supranational social‐policy agencies to be concerned not only about their moral reputation but also technical.

Although the technical dimension is also relevant for agencies dealing with economic policies, we expect economic agencies to focus more on the performative and legal‐procedural aspects compared to social‐policy agencies. We expect this because performative capacity is at the nucleus of EU economic agencies' mandates (i.e., to deliver effective regulatory outputs) by, for example, facilitating a smooth exchange of goods between Member States that is expected to result in positive outcomes (e.g., economic growth). Supranational agencies contributing to the EU internal market have to demonstrate their capacity to take effective actions that significantly affect powerful audiences, for example, Member States, the regulated industry. For instance, supranational agencies have to prove their capacity to coerce Member States (e.g., countries that do not follow commonly agreed standards created to assist the common market harmonization processes) and the regulated industry (e.g., banks that violate supranational rules). Given their core obligations to assist the functioning of the EU market integration, agencies contributing to economic policies are expected to send stronger performative capacity signals (compared to social‐policy agencies) and focus on displaying sufficient strength, assertiveness, and “the ability to intimidate” powerful audiences (Carpenter, 2010, p. 46). Furthermore, we expect the focus on the performative dimension to go hand‐in‐hand with the emphasis on the legal‐procedural aspects of economic‐policy agencies activities. We expect this because in order to sway powerful audiences to respect EU rules and standards, economic‐policy agencies have to prove that they themselves arrive at the common rules following due processes. This is particularly relevant in light of the recent crises in the European economic/financial sector that originated from the performative failures and legal‐procedural misconduct (e.g., the violation of rules and flouting due processes). To that end, agencies contributing to economic policies are expected to emphasize the performative and legal‐procedural aspects of their core activities to regain/enhance a positive organizational reputation.

Hypothesis 3a/3b *Agencies contributing to social policies are more likely to focus on technical/moral‐reputation aspects than agencies contributing to economic policies*.

Hypothesis 4a/4b *Agencies contributing to economic policies are more likely to focus on performative/procedural‐reputation aspects than agencies contributing to social policies*.

How agencies manage their organizational reputation is anticipated to be affected not only by their reputation uniqueness but also by reputational threats. Reputational threats are defined as “challenges that pose a threat to the agency's established reputation, consisting of external opinions and allegations from (a) particular audience(s)” (Gilad et al., 2013, p. 452). The reputational threats rise when the internal day‐to‐day activities and decision‐making processes start to be regarded as critically relevant to external audiences (i.e., when the glare of public attention and political scrutiny is high). An increased level of audiences' awareness about an agency results in augmented conflicting demands that create a difficult situation for agencies because they have to balance between incompatible expectations (Carpenter & Krause, 2012). This, in turn, leads to the unattainable task of cultivating a good reputation in the eyes of conflicting audiences. As a result, higher reputational threats are expected to urge agencies to cultivate their intermediate responses more carefully by producing different blame‐avoidance reactions by strategically combing the reciprocity of reputational dimensions.

Higher reputational threats are expected to encourage agencies to become more *entrepreneurial* and expand their reputational repertoire by focusing not only on output‐oriented legitimacy (i.e., performative and/or technical reputation) but also on the procedural and/or moral aspects (Busuioc & Rimkutė, 2019a). In other words, greater reputational threats are anticipated to urge EU agencies to depart from their declared raison d'être (i.e., pronounced emphasis on the credible, effective, efficient, technical, and/or scientific aspects of agency conduct). This is because greater reputational threats entail serious organizational risks that can damage agencies' legitimacy and image in the polity. As a result, in view of high reputational threats, reliance on output‐oriented legitimacy sources is a necessary but not sufficient means to gain, maintain, or enhance a positive organizational reputation.

More specifically, *different degrees of reputational threats are* expected to lead to diverse response strategies: EU agencies performing in contexts containing higher reputational threats have to (re)establish confidence; demonstrate that they care about those affected by regulations; and spend significant time and effort on (re)gaining trust by emphasizing their organizational transparency, openness, and inclusiveness. This is because agencies strive to balance between pursuing good organizational reputations and receptiveness to relevant public demands (Gilad, 2015; Gilad & Alon‐Barkat, 2018; Maor & Sulitzeanu‐Kenan, 2013). Public organizations aim at survival and the legitimation of their roles and activities, which, in turn, make them more sympathetic to prevailing public concerns (Carpenter, 2010). Thus, one would expect that agencies facing *higher reputational threats* would focus on demonstrating that they are empathetic to the concerns of those affected by their conduct.

Furthermore, higher reputational threats are expected to draw agencies' attention to the communications that emphasize their due processes and attempts to convince those who are closely observing their conduct that proper and fair procedures were adhered to. Attentively managing (legal‐)procedural aspects of the agency's conduct are argued to be efficient strategies for fostering legitimacy (Carpenter, 2010) because of the pervasive perception that openness increases the legitimacy of the decision‐making process (Worthy, 2010). Organizations can advance their legitimacy by carefully justifying the verdicts taken in the decision‐making process. On the other hand, agencies facing lower reputational threats are expected to remain true to the regulatory state discourse by exclusively focusing on output‐oriented legitimation sources, or their science‐based and results‐oriented conduct.

Hypothesis 5a/5b *Agencies facing higher reputational threats are more likely to emphasize their procedural/moral‐reputation than agencies facing lower reputational threats*.

Hypothesis 6a/6b *Agencies facing lower reputational threats are more likely to emphasize their technical/performative‐reputation than agencies facing higher reputational threats*.

## 4 RESEARCH DESIGN

To test the hypotheses, the study draws on the most recent communications of 45 EU agencies and bodies. Their communications allow us to not only capture which organizational‐reputation aspects EU agencies focused on when presenting themselves to relevant audiences but also empirically observe how agencies attempted to bridge a gap between the images they have and the images they would like to attain by emphasizing certain aspects of their current day‐to‐day activities and future strategies.

The empirical focus was on information that agencies choose to provide on the “about us” section of their websites; annual reports (2016) summarizing their core activities; and multiannual programming documents (2017–2019) that communicate future goals, strategies, and plans. We compiled and analyzed a document ranging from 150 to 400 pages for each agency. These documents cover a full range of communications that agencies publish to “speak” to a wide range of audiences: information on the Web sites is aimed at the media and citizens, while annual reports and programming documents target professional audiences (e.g., EU institutions, NGOs, the industry). The selected documents contain detailed and rich information relating to the agencies' core priorities, objectives, and actual accomplishments, which correspond with their formal mandates and are drafted in line with their strategy documents.

### 4.1 Operationalization

To empirically observe the four dimensions of organizational reputation, the study relies on the Busuioc and Rimkutė's (2019a) measurement tool. Relying on Carpenter's (2010) definitions, the authors deductively derived and empirically tested the keywords characterizing each dimension (technical, performative, procedural, moral). Although the list of keywords is not exhaustive, the strength of this measurement is its strict reliance on the relevant theoretical concepts and its aim to derive keywords that precisely tap into one of the four categories. However, the authors acknowledged that, although keywords provide relevant insight on how frequently agencies refer to technical, performative, procedural, and/or moral vocabulary, this approach does not allow to capture more nuanced messages that agencies convey to diverse and conflicting audiences.

Regarding the operationalization of explanatory factors, the study draws on the Majone's (1997) definition and reasoning about the European regulatory state and EU agencies. In this study, regulatory agencies include all agencies that follow the Majone's proposed logic of “regulation by information” (i.e., agencies in charge of information gathering, technical advice, standard setting, or [quasi‐]decision‐ or rule‐making tasks), whereas nonregulatory agencies comprise those possessing executive and coordination tasks.^2^ Furthermore, following Majone's (1997) argumentation, we classified agencies contributing to environmental, health (including such sectors as food, chemicals, and medicines/drugs), and broader safety and security issues as social‐policy agencies, whereas we classified agencies assisting EU institutions and Member States in proper functioning of the European single market as contributing to economic policies.^3^

To identify which agencies have received high or low reputational threats, we counted the EP's questions that directly mention the name of an EU agency. We analyzed parliamentary questions raised in 2015 to account for EU agency communication practices in 2016 (annual reports and programming documents issued in 2016). Font and Pérez Durán (2016) found that written EP questions were concentrated on agencies that generate more media attention. Therefore, we regarded the number of parliamentary questions directly addressing a specific EU agency as a proxy of the agency's exposure to multiple competing audiences that impose conflicting expectations on the agency and, in so doing, higher reputational threats. In counting parliamentary questions that target EU agencies, we found three agencies that have received more attention from the EP in 2015: Frontex, Europol, and the European Food Safety Authority (EFSA) (slightly above/below 100 parliamentary questions). The EP has also directly mentioned the following agencies but to a lesser extent (over 10 mentions per year): aviation safety (EASA), asylum support (EASO), banking (EBA), disease prevention (ECDC), environmental protection (EEA), medicines (EMA), drugs (EMCDDA), securities and market (ESMA), working conditions (Eurofound), judicial cooperation (Eurojust), and human rights (FRA). The remaining agencies either have not been mentioned at all or received only a couple of mentions; therefore, we classified these as agencies facing low reputational threats.

## 5 EMPIRICAL ANALYSIS

For our empirical analysis, we used WordStat, a content‐analysis and text‐mining software that is suitable for handling large amounts of word‐based information, to analyze the rich textual data. We analyzed the data retrieved from WordStat—the frequency/rate/percentage of the keywords used by an agency in its communications—using R.

### 5.1 How do EU agencies build organizational reputation?

An initial overview of the communications of EU agencies shows that explicit references to performative and technical reputation dimensions are most commonly used, whereas procedural and moral reputation aspects are addressed to a lesser extent (Table 2). This empirical observation is in line with the expectations regarding the regulatory state's discourse. EU agencies' legitimation efforts are focused on output‐oriented legitimacy, which encompasses both performative and technical reputations, whereas a reliance on input‐legitimacy is less pronounced.

**Table 2:** Frequency/percentage of organizational reputation dimensions

Reputation dimension	Frequency (% of words)	Percentage of total words
Performative	51,735 (41.69)	2.73
Technical	47,381 (38.18)	2.50
Procedural	18,387 (14.82)	0.97
Moral	6,593 (5.31)	0.35
Total	124,096 (100)	6.55

**Figure 1:**
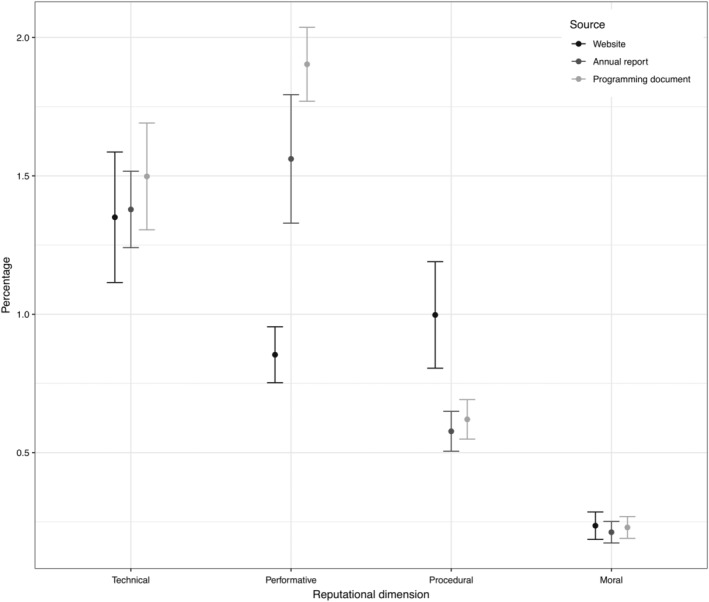
Comparison between different communication sources

The comparison between the different sources of communication (websites/annual reports/programming documents) shows that there are no significant differences in how agencies cultivate their technical and moral reputations across different communication channels (Figure 1). Throughout the three communication sources agencies consistently emphasize the relevance of their technical/professional contribution to the European regulatory state, whereas they remain modest in underscoring the wider moral implications of their activities. However, agencies tend to focus significantly more on conveying performative messages in programming documents compared to other sources, whereas they use websites to send strong procedural appropriateness signals. These findings support the theoretical claim that agencies are selective and strategic in how they present themselves to different audiences, that is, with programming documents and annual reports agencies target professional audiences that care about their performance indicators, whereas they choose websites—that cover a more diverse set of stakeholders including EU citizens and the media—to signal the alignment of their day‐to‐day activities with the procedural requirements.

**Figure 2:**
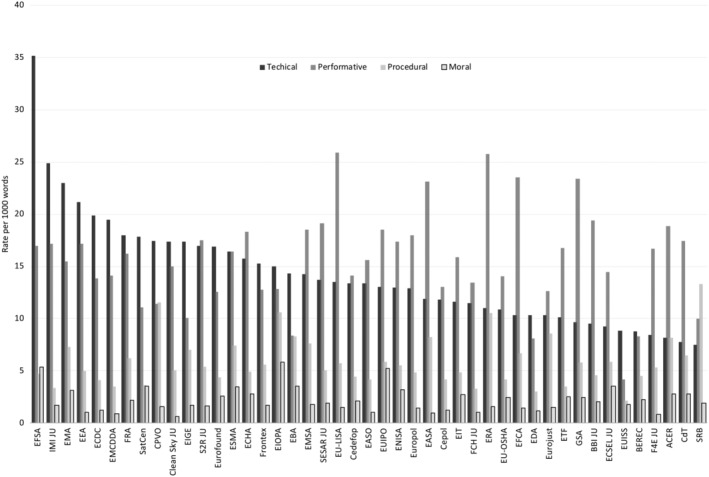
Rate of reputational dimension per 1,000 words. ACER, Agency for the Cooperation of Energy Regulators; BBI JU, Bio‐Based Industries Joint Undertaking; BEREC, Office of the Body of European Regulators for Electronic Communications; CdT, Translation Centre for the Bodies of the European Union; CEDEFOP, European Centre for the Development of Vocational Training; CEPOL, European Police College; CLEAN SKY JU, Clean Sky Joint Undertaking; CPVO, Community Plant Variety Office; EASA, European Aviation Safety Agency; EASO, European Asylum Support Office; EBA, European Banking Authority; ECDC, European Centre for Disease Prevention and Control; ECHA, European Chemicals Agency; ECSEL JU, Electronic Components and Systems for European Leadership Joint Undertaking; EDA, European Defense Agency; EEA, European Environment Agency; EFCA, European Fisheries Control Agency; EFSA, European Food Safety Authority; EIGE, European Institute for Gender Equality; EIOPA, European Insurance and Occupational Pensions Authority; EIT, European Institute of Innovation and Technology; EMA, European Medicines Agency; EMCDDA, European Monitoring Centre for Drugs and Drug Addiction; EMSA, European Maritime Safety Agency; ENISA, European Union Agency for Network and Information Security; ERA, European Railway Agency; ESMA, European Securities and Markets Authority; ETF, European Training Foundation; EUIPO, European Union Intellectual Property Office; EUISS, EU Institute for Security Studies; EU‐LISA, EU Agency for large‐scale IT systems; EU‐OSHA, EU Agency for Safety and Health at Work; EUROFOUND, European Foundation for the Improvement of Living and Working Conditions; EUROJUST, EU's Judicial Cooperation Unit; EUROPOL, European Police Office; F4E JU, European Joint Undertaking for ITER and the Development of Fusion Energy; FCH 2 JU, Fuel Cells and Hydrogen Joint Undertaking; FRA, EU Agency for Fundamental Rights; FRONTEX, EU Agency for the Management of Operational Cooperation at the External Borders; GSA, European GNSS Agency; IMI JU, Innovative Medicines Initiative 2 Joint Undertaking; SatCen, EU Satellite Centre; S2R JU, Shift2Rail Joint Undertaking; SESAR JU, SESAR Joint Undertaking; SRB, Single Resolution Board

**Figure 3:**
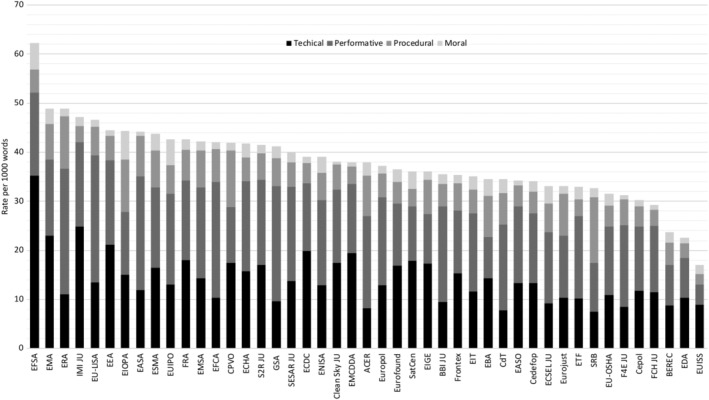
Combination of the technical, performative, procedural, and moral dimensions per agency

Although an emphasis on technical and performative reputations is featured across all agencies, there is a substantial variance in how supranational authorities manage their reputations and how they combine the four reputational dimensions (Figure 2). EU agencies emphasize different reputational aspects, engage in different combinations of reputational dimensions, and cultivate organizational reputation to various degrees. To illustrate, Figure 3 shows that agencies differ in terms of how they combine a reciprocity of reputational dimensions. For instance, EFSA is the most strategic in cultivating its reputation, that is, it uses the most keywords (62.23 per 1,000 words) to present itself to external audiences, whereas the Institute for Security Studies (EUISS) refers to organizational reputation keywords relatively seldom (16.99 per 1,000 words). Nevertheless, one prominent tendency is evident: The technical and performative dimensions represent the core basis of reputation‐management efforts.

### 5.2 Do reputation uniqueness and reputational threats account for the diverse reputation‐management strategies across EU agencies?

We conducted an agency‐level analysis to examine how the management of organizational reputation varies across agencies that possess different reputation uniqueness and are exposed to varying degrees of reputational threats. The study considered the differences between regulatory and nonregulatory agencies, agencies possessing social‐policy and economic‐policy competencies, as well as the agencies facing high and low reputational threats. To examine whether these between‐group differences were substantial, we conducted a *t* test to determine if mean differences (rate of technical, performative, procedural, or moral dimension per 1,000 words) were statistically significant.

**Figure 4:**
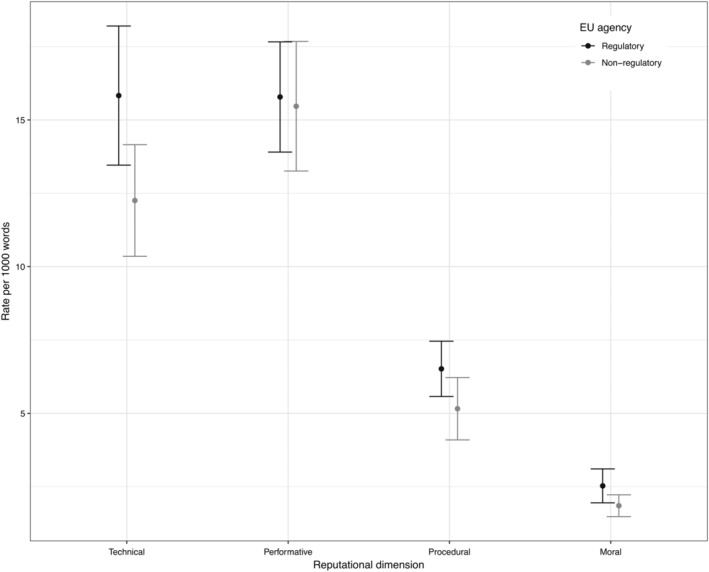
Regulatory agencies versus nonregulatory agencies

The results suggest that regulatory agencies are more strategic in how they cultivate their reputations (i.e., they paid more attention to all dimensions) compared to nonregulatory agencies (Figure 4). On average, regulatory agencies focused more on the technical dimension (*M* = 15.83, *SD* = 5.62) than nonregulatory agencies (*M* = 12.25, *SD* = 4.18), *t*(41.99) = 2.44, *p* = .019. Our results suggest that regulatory agencies also focused more on the moral dimension (*M* = 2.53, *SD* = 1.37) than executive or coordination agencies (*M* = 1.85, *SD* = 0.82), *t*(38.23) = 2.04, *p* = .048. They also scored higher on the procedural dimension (*M* = 6.52, *SD* = 2.23) compared to nonregulatory agencies (*M* = 5.16, *SD* = 2.34), *t*(41.63) = 1.99, *p* = .053. Finally, empirical evidence showed that agencies with regulatory tasks tended to focus a bit more on the performative dimension (*M* = 15.78, *SD* = 4.45) compared to nonregulators (*M* = 15.47, *SD* = 4.85); however, the difference was not significant, *t*(40.97) = 0.23, *p* = .821. While we expected EU regulatory agencies to focus more on technical, procedural, and moral dimensions (2a/2b/2c), we did not anticipate them to manage their performative dimension to the same extent as their nonregulatory counterparts (Hypothesis 1a). This suggests that performative reputation is at the core of both regulatory and nonregulatory agencies' efforts, that is, both emphasize their capacity to attain goals set in their formal mandates by delivering effective and efficient regulatory outputs.

**Figure 5:**
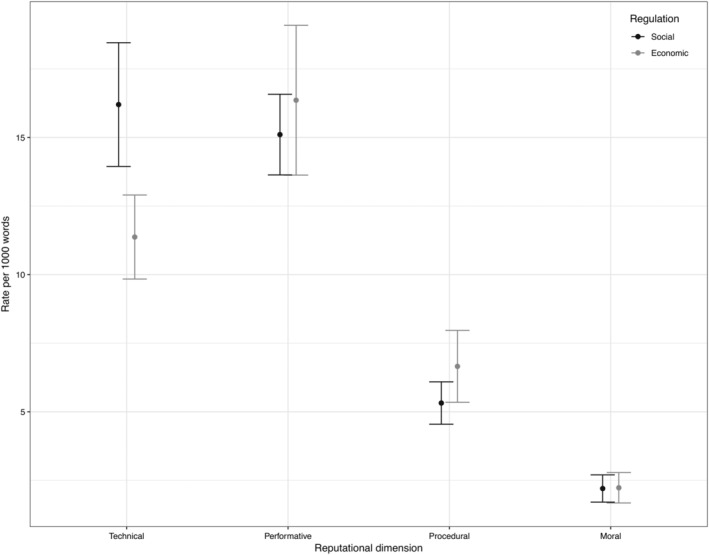
Social‐policy agencies versus economic‐policy agencies

The content analysis on the communications of EU agencies showed some statistically significant differences in how agencies dealing with social and economic policies manage their organizational reputation (Figure 5). On average, agencies contributing to social policies paid significantly more attention to their technical reputation (*M* = 16.20, *SD* = 5.59) than agencies concerned with economic policies (*M* = 11.37, *SD* = 3.18), *t*(40.90) = 3.67, *p* = .000, whereas economic agencies focused more on the procedural aspects of their reputation (*M* = 6.66, *SD* = 2.72) compared to their counterparts dealing with social policies (*M* = 5.32, *SD* = 1.91), *t*(30.50) = −1.84, *p* = .076. Furthermore, economic‐policy agencies (*M* = 16.36, *SD* = 5.67) focused more on performative reputation than social‐policy agencies (*M* = 15.11, *SD* = 3.64); however, the difference was not statistically significant, *t*(28.63) = −0.85, *p* = .405. The results indicate that only hypothesis 3a receives substantial empirical support, that is, agencies contributing to social policies are more likely to focus on technical‐reputation aspects than agencies contributing to economic policies. Although the findings are in line with the Hypotheses 4a/4b—agencies contributing to economic policies are more likely to focus on performative/procedural‐reputation aspects than agencies contributing to social policies—the empirical data indicate statistically insignificant differences between social‐ and economic‐policy agencies when it comes to the performative and procedural reputation‐management strategies. Furthermore, the expectation that social‐policy agencies focus on moral reputation more than economic‐policy agencies also does not hold true (3a/3b). Economic (*M* = 2.23, *SD* = 1.15) and social‐policy agencies (*M* = 2.20, *SD* = 1.23) scored similarly on the moral dimension, *t*(40.34) = −0.08, *p* = .940, which is surprising given the expectation that moral implications of social‐policy agencies' outputs should be at the heart of their reputation uniqueness.

**Figure 6:**
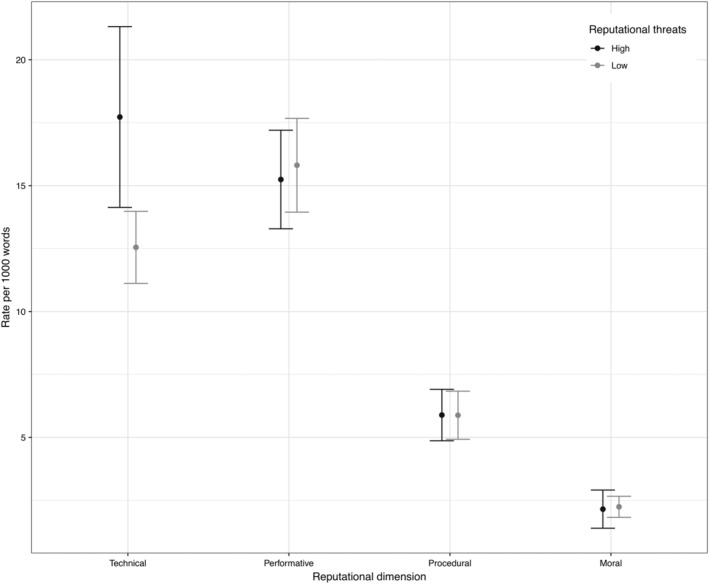
High versus low reputational threats

Contradictory to the theoretical expectation that agencies facing higher reputational threats were more likely to emphasize their procedural or moral reputation (Hypotheses 5a/5b) and agencies facing lower threats would emphasize the technical or performative dimensions (6a/6b), the empirical findings showed that, on average, agencies receiving higher reputational threats were more likely to emphasize their technical reputation (*M* = 17.73, *SD* = 6.22) compared to agencies facing lower threats (*M* = 12.55, *SD* = 3.90), *t*(17.78) = 2.87, *p* = .01. In line with our theoretical expectation, agencies experiencing lower reputational threats tended to focus more on the performative dimension (*M* = 15.81, *SD* = 5.08) than agencies facing higher threats (*M* = 15.24, *SD* = 3.39); however, the difference was not statistically significant, *t*(36.45) = −0.44, *p* = .662 (Figure 6). The results, however, suggest that higher reputational threats—measured by the number of parliamentary questions mentioning EU agencies—do not encourage agencies to cultivate moral reputation (*M* = 2.15, *SD* = 1.31) more than agencies experiencing lower threats (*M* = 2.24, *SD* = 1.14), *t*(22.21) = −0.2, *p* = .844. In a similar vein, higher reputational threats did not affect the patterns of procedural reputation management: agencies with high threats (*M* = 5.89, *SD* = 1.77) versus agencies with low threats (*M* = 5.88, *SD* = 2.61), *t*(35.96) = 0.01, *p* = .991.

## 6 DISCUSSION

The results suggest that the general reputation‐management trends encountered in EU agencies' communications are consistent with well‐established and enduring claims that the EU regulatory state is predominantly focused on output‐legitimation strategies (Majone, 1999; Scharpf, 1999), encompassing both technical and performative aspects. The findings support the claim that EU agencies are legitimated through the delivery of sound outputs, which are deemed to be rooted in expertise and are meant to contribute to the effectiveness of the EU's policy outcomes. The EU agencies' technical characters are expected to have direct consequences for their outputs: “policies…are basically legitimated by results, and hence may be delegated to such [nonmajoritarian] institutions” (Majone, 1998, p. 28).

In contrast, the reliance on input‐oriented legitimation sources (i.e., procedural and moral reputations) is trivial in EU agencies' communications. The moral dimension is empathized the least by EU agencies, and this remains constant across all 45 agencies and bodies. The findings support the mainstream claim that the European regulatory state is marked by a strong focus on results‐ or evidence‐centered reasoning when communicating about roles and activities, whereas the moral dimension—“Does the organization exhibit compassion for those adversely affected by its decisions …? ” (Carpenter, 2010, p. 46)—remains at the realm of national regulators that function closer to those affected by regulations, for example, consumers (see Rimkutė, 2018). However, future research could empirically test whether this claim holds and attempt to answer the following questions: Do national regulators draw more on the moral or procedural aspects of organizational reputation than their supranational counterparts? How do the reputation‐management efforts of supranational agencies compare to their national counterparts?

Although supranational agencies are skewed toward the output‐based sources of legitimation, the systematic mapping of organizational‐reputation‐management patterns across all EU agencies has illustrated that agencies vary in how they present themselves to relevant audiences. We have learnt that EU agencies emphasize different reputational aspects, employ different combinations of reputational dimensions, and cultivate their organizational reputation to different degrees. Furthermore, we have found support to the seminal reputation literature claim stating that reputational considerations drive public organizations' behavior (Carpenter, 2010). Our findings suggest that declared regulatory roles make a difference in how EU agencies portray themselves to external audiences that judge and validate their organizational conduct. The empirical results reveal that regulatory agencies are more astute in their reputation management compared to their nonregulatory counterparts. Regulatory agencies utilize a more diverse set of reputational strategies and, on average, tend to emphasize the technical, procedural, and moral dimensions more compared to their nonregulatory counterparts. This finding suggests that agencies acting as fiduciaries enhancing a credible commitment are more strategic than agencies following the efficiency‐based logic of delegation (Majone, 2001). In other words, EU regulatory agencies care more about the reciprocity of their reputational dimensions. This finding is in line with the most recent scholarship arguing that EU regulatory agencies have become more entrepreneurial over time as they invest more efforts in harvesting various aspects of their bureaucratic reputations (Busuioc & Rimkutė, 2019a).

Our empirical results suggest that agencies contributing to social policies are more likely to show a superior track record in terms of their technical and scientific conduct compared to agencies contributing to economic policies in the EU regulatory state. In addition, our findings reveal that agencies operating in the area of economic policies work harder on building a strong reputation for procedural appropriateness by emphasizing their adherence to fair procedures and by demonstrating that their day‐to‐day activities are comme il faut in terms of commonly accepted rules. These findings are in line with the theoretical expectation that agencies engage in a selective response—for example, blame‐avoidance games based on reputational considerations—to limit the types of failures that are most threatening to their survival (Maor & Sulitzeanu‐Kenan, 2013; Weimer, 2006). In light of the recent economic crises and regular scandals in the social‐regulation domain (e.g., BSE scandal, immigration crisis), agencies in those respective fields are responsive to the reputational threats they face, that is, fear to appear as incapable in delivering credible solutions rooted in sound evidence and rigorous scientific methodologies (for agencies working in social‐policy domains) *or* fear to appear as negligent to the rules and procedures that were established to prevent future crises in the economic‐policy domain.

Last but not least, we have learnt that EU agencies facing higher reputational threats are more likely to focus on the technical aspects of their reputation compared to agencies that face lower threats. The empirical results imply that agencies experiencing multifaceted expectations originating from conflicting audiences tend to revert to their declared raison d'être (i.e., the consequentialist perspective on political rule that is legitimized based on a reliance of the best available evidence). This finding is in line with the study of Maor et al. (2012) suggesting that agencies choose to be responsive to external signals that target their core functions because those signals are the most threatening to the reputational uniqueness of an agency. We have learnt that supranational agencies choose to address multiple audiences and resist aggressive attacks by authoritative audiences by communicating their adherence to the highest technical/scientific standards. This finding suggests that higher reputational threats encourage EU agencies to fight external attacks by referring to their technical conduct (e.g., scientific gold standard), which is regarded as a powerful reputation‐protection mechanism in blame‐avoidance and diffusion games (Maor, 2007; Rimkutė, 2018). However, future research should focus on uncovering causal mechanisms behind the relationship between reputational threats and reputation‐management strategies. A good starting point could be Maor et al. (2012) and Gilad et al. (2013) studies suggesting that the reputational strengths or vulnerabilities of an agency form the basis for its reputation‐management strategy.

## 7 CONCLUSION

The organizational reputation theory argues that public organizations play different reputation‐promotion games. Organizations have a repository of strategies they combine to deal with expectations and attacks from conflicting external audiences (Carpenter, 2010). However, reputation literature has not been used to explain the diverse patterns of EU agencies' communications. To address these questions, this study has drawn on a quantitative analysis of 45 EU agencies' communications to determine which dimensions of organizational reputation are relevant to supranational agencies, how the repository of reputation cultivation strategies varies across agencies, and whether the variance in reputation‐management patterns corresponds with the variance in reputation uniqueness or/and reputational threats. In doing so, this study has bridged two literature streams—organizational reputation and supranational governance—by addressing critical but, so far, underexplored questions in the context of the European regulatory state. Such contribution not only provides novel insights into the context of supranational regulatory governance but also suggests wider implications into the theoretical development of the reputation scholarship by theorizing how reputation uniqueness and reputational threats guide organizations' reputation‐management strategies deployed by public organizations operating in complex multilevel political systems to legitimize themselves vis‐à‐vis multifaceted audiences.

This quantitative study provided the first systematic mapping of how reputational considerations are managed across EU agencies and, in so doing, has opened many avenues for future research. Although the findings suggest that, overall, EU agencies tend to rely on the output‐based‐legitimation sources, the study found that reputation uniqueness accounts for reputation‐management differences across EU agencies; that is, the results suggest that regulatory agencies care more about the reciprocity of their reputational dimensions by emphasizing the technical, procedural, and moral dimensions more compared to their nonregulatory counterparts, whereas social‐policy agencies foster their technical reputation to a larger extent than economic‐policy agencies. Furthermore, study has found that reputational threats encourage agencies to safeguard their core organizational values, which are related to the long‐established reasoning that the EU regulatory state relies on output‐oriented legitimation sources; that is, we find that agencies facing higher reputational threats focus on their avowed raison d'être, that is, technical reputation.

However, we still lack an understanding of the types of reputational threats that urge agencies to be more responsive to prevailing public pressures. For instance, when and under what conditions do agencies diverge from their avowed raison d'être? This study only looked at the multiplicity of conflicting expectations and public attention to an agency. It did not explore how the nature and type of reputational threats or different constellations of audiences affect agency behavior. Future research could, for example, explore how the valence of public allegations alters reputation‐management strategies, which types of reputational threats (targeting technical conduct, performative capacities, procedural appropriateness, or moral image) are relevant to agencies, and how agencies prioritize and respond to a particular type or combination of reputational threats. In addition, this study mapped out the differences between reputation‐management strategies across agencies, future research could focus on how reputation is managed over time/across different communication channels and identify underlying causal mechanisms behind the relationship between reputational threats and reputation‐management strategies.

## CONFLICT OF INTEREST

The author declares no potential conflict of interest.

## ENDNOTES

1 At the EU level, social‐policy agencies refer to the agencies working in domains such as environment, food safety, pharmaceuticals, chemicals, whereas economic‐policy agencies include not only economic and financial regulators but also agencies contributing to the smooth functioning of the EU single market (e.g., the European Railways Agency; the European Fisheries Control Agency) (see Majone, 1997; endnote 3). It is important to emphasize that as the EU is not a welfare state (i.e., it plays a limited role in redistributive social policies), its social‐policy agencies are different compared to their national counterparts; that is, their reputational uniqueness is focused on providing reliable (scientific) evidence to political superiors that make political decisions, which in turn has implications on our expectations and causal reasoning behind the hypotheses.

2 Regulatory agencies: ACER, BEREC, CEDEFOP, CVPO, EASA, EBA, ECDC, ECHA, EEA, EFCA, EFSA, EIGE, EIOPA, EMA, EMCDDA, EMSA, ENISA, ERA, ESMA, EUIPO, EU‐OSHO, EUROFOUND, FRA, Frontex. Nonregulatory agencies: BBI JU, CdT, CEPOL, CLEAN SKY JU, EASO, ECSEL JU, EDA, EIT, ETF, EUISS, EU‐LISA, EUROJUST, EUROPOL, F4E JU, FCH 2 JU, GSA, IMI JU, SatCen, SESAE JU, S2R JU, SRB. Please see endnote V for full names of the agencies.

3 Agencies contributing to social policies: CEDEFOP, CEPOL, EASA, EASO, ECDC, ECHA, EEA, EFSA, EIGE, EIOPA, EMA, EMCDDA, EMSA, ENISA, ETF, EUISS, EU‐OSHA, EUROFOUND, EUROJUST, EUROPOL, FRA, Frontex, IMI JU, SatCen, SESAE JU, S2R JU. Agencies contributing to economic policies: ACER, BEREC, BBI JU, CdT, CLEAN SKY JU, CVPO, EBA, ECSEL JU, EDA, EFCA, EIT, ERA, ESMA, EUIPO, EU‐LISA, F4E JU, FCH 2 JU, GSA, SRB.
